# An updated list of the *Culicoides* (Diptera, Ceratopogonidae) fauna from Ecuador[Fn FN1]

**DOI:** 10.1051/parasite/2022061

**Published:** 2022-12-23

**Authors:** Juan D. Mosquera, Sonia Zapata, Gustavo Spinelli, Moises Gualapuro, Renato León, Denis Augot

**Affiliations:** 1 Instituto de Microbiología, Colegio de Ciencias Biológicas y Ambientales, Universidad San Francisco de Quito, Diego de Robles and Pampite 170901 Quito Ecuador; 2 Estación de Biodiversidad Tiputini, Colegio de Ciencias Biológicas y Ambientales, Universidad San Francisco de Quito USFQ Quito Ecuador; 3 División Entomología, Museo de La Plata, Paseo del Bosque 1900 La Plata Argentina; 4 Instituto de Limnología “Dr. Raúl A. Ringuelet”, Consejo Nacional de Investigaciones Científicas y Técnicas de la Argentina, Universidad Nacional de La Plata Boulevard 120 y 62 1900 La Plata Buenos Aires Argentina; 5 UscVecpar, ANSES-LSA-EA7510, SFR Cap Santé, Université de Reims Champagne-Ardenne 51 rue Cognacq-Jay 51096 Reims Cedex France; 6 Anses, INRAe, ENVA, UMR-BIPAR, Laboratoire de Santé Animale 14 rue Pierre et Marie Curie 94701 Maisons-Alfort Cedex France

**Keywords:** Biting midges, New records, Diversity, *Culicoides*, Ecuador

## Abstract

An updated list of biting midges of the genus *Culicoides* inhabiting Ecuador is provided. Entomological investigations were carried out from July 2010 to May 2019 using CDC light traps in three Ecuadorian regions (Amazon basin, Andean (foothills and highlands) and Pacific Coast). A total of 12,073 *Culicoides* specimens from seven subgenera and nine species groups were collected. More species and higher variation were found in the Amazon basin than in either of the Andes regions or coastal sites. A total of 53 species were identified. Of these, 15 are herein reported as new species records for Ecuador: *Culicoides acotylus* Lutz, *C. aitkeni* Wirth & Blanton, *C. benarrochi* Ortiz & Mirsa, *C. carvalhoi* Wirth & Blanton*, C. freitasi* Wirth & Blanton, *C. ginesi* Ortíz, *C. lopesi* Barretto*, C. lyrinotatus* Wirth & Blanton*, C. profundus* Santarém, Felippe-Bauer & Trindade, *C. pseudoreticulatus* Santarém, Felippe-Bauer & Castellón*, C. quasiparaensis* Clastrier, *C. vernoni* Wirth & Blanton, *C. youngi* Wirth & Barreto and two new species. Our results show that the updated list of the Ecuadorian *Culicoides* fauna comprises 70 species. This inventory highlights the presence of species that have been incriminated as vectors of disease elsewhere in animals and humans, mainly *C. insignis* and *C. paraensis*.

## Abstract (fr)

Une liste actualisée des *Culicoides* vivant en Équateur est fournie. Des enquêtes entomologiques ont été menées de juillet 2010 à mai 2019 à l’aide de pièges lumineux CDC dans trois régions équatoriennes (le bassin amazonien, les Andes (piémont et hautes terres) et la côte Pacifique). Au total, 12 073 spécimens de *Culicoides* de sept sous-genres et neuf groupes d’espèces ont été collectés. Plus d’espèces et une variation plus élevée ont été trouvées dans le bassin amazonien que dans les régions des Andes ou les sites côtiers. Au total, 53 espèces ont été identifiées. Parmi celles-ci, 15 sont signalées ici comme nouvelles espèces pour l’Équateur : *Culicoides acotylus* Lutz, *C. aitkeni* Wirth & Blanton, *C. benarrochi* Ortiz & Mirsa, *C. carvalhoi* Wirth & Blanton, *C. freitasi* Wirth & Blanton, *C. ginesi* Ortíz, *C. lopesi* Barretto, *C. lyrinotatus* Wirth & Blanton, *C. profundus* Santarém, Felippe-Bauer & Trindade, *C. pseudoreticulatus* Santarém, Felippe-Bauer & Castellón, *C. quasiparaensis* Clastrier, *C. vernoni* Wirth & Blanton, *C. youngi* Wirth & Barreto et deux nouvelles espèces. Nos résultats montrent que la liste actualisée de la faune culicoïde équatorienne comprend 70 espèces. Cet inventaire met en évidence la présence d’espèces qui ont été incriminées ailleurs comme vecteurs de maladies chez l’animal et l’homme, principalement *C. insignis* et *C. paraensis*.

## Introduction

Biting midges of the genus *Culicoides* Latreille (Diptera: Ceratopogonidae) are the smallest (1–3 mm) of the hematophagous flies and are broadly distributed throughout the world [6]. These insects are vectors of livestock viruses, including bluetongue virus (BTV), African horse sickness virus (AHSV), Akabane virus (AKAV), Epizootic hemorrhagic disease virus (EHDV) [28], Schmallenberg virus [22], as well as Oropouche virus (OROV), which causes clinical symptoms such as fever, headache, muscle and joint pain, skin rash, meningitis and/or encephalitis in humans [36].

Both BTV and OROV are encountered in South America [28]. Based on vector distribution and environmental conditions, the epidemiology of BTV can be categorized into three zones: endemic (South America north of the Tropic of Capricorn), epidemic (south of the Tropic of Capricorn to Uruguay) and incursion zone (South of Uruguay) [37]. In the Neotropical region, *Culicoides insignis* Lutz and to a lesser extent *C. filarifer* Hoffman, *C. pusillus* Lutz and *C. trilineatus* Fox [24] are suspected to transmit BTV, while *C. paraensis* (Goeldi) is a proven vector of OROV [28].

In Ecuador, OROV has been reported in the Pastaza province in the Ecuadorian Amazon basin [26] and in the Esmeraldas province in the coastal region [58]. Similarly, BTV has been reported: in cattle in El Oro Province in southern Ecuador [23], as well as in Pichincha, Napo, Esmeraldas and Manabi Provinces [11, 50]; in sheep in West of Ecuador [27] and in Pichincha Province [30]. EHDV is present in Pichincha, Napo, Esmeraldas Provinces [50]. In contrast, BTV and epizootic hemorrhagic disease virus (EHDV) are not present in cattle in the Galapagos Islands [51].

Inventories of *Culicoides* have been carried out in the Neotropical region since the middle of the 20th century. The studies carried out by O.P. Forattini and W.W. Wirth and collaborators, and the standardization of morphological characters used to describe *Culicoides* species led to the production of identification aids and dichotomous keys [17, 21, 35, 43, 53, 56].

Moreover, in the list of the biting midges of the World [7], more than 300 new species were described from the Neotropical region between 1853 and 2016.

Ortíz and León [34] first investigated the genus in Ecuador, with the description of five new species (*C. balsapambensis* Ortíz & Léon*, C. camposi* Ortíz & León, *C. contubernalis* Ortíz & León*, C. insinuatus* Ortíz & León and *C. limonensis* Ortíz & León). Subsequently, a series of publications by several authors contributed to the rise in the number of species in Ecuador to 55 (Table 1) [1, 15, 17, 47, 49, 52, 55, 57].

**Table 1:** *Culicoides* species list for Ecuador.

Subgenus	Species (synonym)	Trap locality	References
*Anilomyia* Vargas, 1960	***C. efferus*** Fox, 1952	(Ti)	[56]
	***C. metagonatus*** Wirth & Blanton, 1956	(Pa, Ti2)	[49, 56]
*Avaritia* Fox, 1955	***C. pusillus*** Lutz, 1913	(PE, SD, Oz)	[17, 52, 54]
*Diphaomyia* Vargas, 1960	***C. freitasi*** Wirth & Blanton 1973	(Ti)	This study
*Haematomyidium* Goeldi, 1905	**C. *debilipalpis*** Lutz, 1913 (*khalafi* Beck, 1957; *ichesi* Ronderos & Spinelli, 1995)		[17, 52]
	***C. equatoriensis*** Barbosa, 1952 (as a variety of *debilipalpis)*	(Ti2)	[49, 56]
	***C. ginesi*** Ortíz, 1951	(Ti, CD, Ti2)	This study
	***C. glabrior*** Macfie, 1940 (as a variety of *debilipalpis) (grahambelli,* Forattini, 1956)	(Ti, CD, Ti2)	[49, 56]
	***C. insinuatus*** Ortíz & Léon, 1955	(CD)	[17, 49, 54, 56]
	***C. limonensis*** Ortíz & Léon, 1955	(Ti2)	[17, 49, 54, 56]
	***C. neoparaensis*** Tavares & Souza, 1978	(Ti)	[13, 49]
	***C. paraensis*** (Goeldi), 1905 (*undecimpunctatus* Kieffer, 1917)	(Ti, CD, Ti2)	[13, 17, 57]
	***C. quasiparaensis*** Clastrier, 1971	(Ti2)	This study
	***C. youngi*** Wirth & Barreto, 1978	(Pa)	This study
*Hoffmania* Fox, 1948	***C. aitkeni*** Wirth & Blanton, 1968	(Ti)	This study
	***C. batesi*** Wirth & Blanton, 1973 (*sanmartini* Wirth & Barreto, 1978)	(PE, Oz, Ti, Ti2)	[47, 49, 56]
	***C. contubernalis*** Ortíz & Léon, 1955 (as a variety of *rozeboomi)*	(Ti)	[16, 34]
	***C. diabolicus*** Hoffman, 1925	(Pa, Ti, PE, Oz, CE, SD, Ti2, Oz2)	[47, 49, 52]
	***C. filariferus*** Hoffman, 1939		[54, 56]
	***C. foxi*** Ortíz, 1950	(Oz, Ti, Oz2)	[47]
	***C. fusipalpis*** Wirth & Blanton, 1973	(Ti2)	[47, 49, 54, 56]
	***C. guttatus*** *(*Coquillet), 1904	(Ti, PE, Oz, Ti2, Oz2)	[17]
	***C. heliconiae*** Fox & Hoffman, 1944 (*rozeboomi* Barbosa, 1947)	(Pa, Ti, PE, Ju)	[16, 49, 56]
	***C. hylas*** Macfie, 1940	(Pa, Ti, CD, PE, Ju, Ti2)	[1]
	***C. insignis*** Lutz, 1913	(Pa, CD, CE, Oz2)	[47]
	***C. lutzi*** Costa Lima, 1937		[17, 47]
	***C. ocumarensis*** Ortíz, 1950	(Oz, SD)	[47, 56]
	***C pseudodiabolicus*** Fox, 1946	(Oz, Oz2)	[47, 54, 56]
	***C. pseudoheliconiae*** Felippe­Bauer, 2008	(Pa, Ti, Oz)	[1]
	***C. tidwelli*** Spinelli, 1993		[47, 49]
	***C. trinidadensis*** Hoffman, 1925 (*oliveri* Fox & Hoffman, 1944; *wokei* Barbosa, 1947 (preoccupied by *Culicoides wokei* Fox, 1947); *diminutus* Barbosa, 1951)		[47, 49]
	***C. verecundus*** Macfie, 1948	(Pa)	[17, 49, 52, 54, 56]
*Macfiella* Fox, 1955	***C. phlebotomus*** (Williston), 1896 (*amazonius* Macfie, 1935)		[49, 52, 56]
*Mataemyia* Vargas, 1960	***C. bricenoi*** Ortíz, 1951	(Ti, Ti2)	[49, 54, 56]
	***C. dicrourus*** Wirth & Blanton, 1955		[49, 56]
*Oecacta* Poey, 1853	***C. alahialinus*** Barbosa, 1952		[17, 49, 52, 56]
	***C. barbosai*** Wirth & Blanton, 1956		[49, 52, 56]
	***C. furens*** (Poey), 1853 (*maculithorax* (Williston), 1896; *dovei* Hall, 1932; *birabeni* Cavalieri, 1966)		[17, 49, 52, 56]
*Psychophaena* Philippi, 1865	***C. venezuelensis*** Ortíz & Mirsa, 1950 (*pictipennis* (Philippi), 1865; *ortizi* Fox, 1952)	(Ur, CD)	[56]
**Species group**			
Acotylus group	***C. acotylus*** Lutz, 1913 (*panamericanus Fox,* 1947)	(Ti)	This study
Carpenteri group	***C. belemensis*** Wirth & Blanton, 1973	(Ti, Ti2)	[1]
	***C. camposi*** Ortíz & Léon, 1955 (*fairchildi* Wirth & Blanton, 1955)	(Ti)	[17, 49, 52, 54, 56]
	***C. carpenteri*** Wirth & Blanton, 1953		[49, 56]
Dasyophrus group	***C. dasyophrus*** Macfie, 1940	(Ti2)	[49, 56]
Eublepharus group	***C. eublepharus*** Macfie, 1948 (*transferrans* Ortíz, 1953)	(Ti)	[17, 49, 52, 54, 56]
	***C. propriipennis*** *Macfie,* 1948		[49, 54, 56]
	***C. rangeli*** Ortíz & Mirsa, 1952 (*donajii* Vargas, 1954; *patulipalpis* Wirth and Blanton, 1959)	(Ti2)	[17, 49, 54, 56]
Fluvialis group	***C. balsapambensis*** Ortíz & Léon, 1955 (as a variety of *C. pifanoi)*	(Ti)	[17, 49, 52, 56]
	***C. castillae*** Fox, 1946 (*gibsoni* Wirth, 1952; *flochabonnenc*i Ortíz & Mirsa, 1952)	(Ti, PE, Oz, Ju, CE, SD, Ti2)	[17, 49, 52, 56]
	***C. leopoldoi*** Ortíz, 1951	(Ti, PE, Ti2)	[56]
	***C. tetrathyris*** Wirth & Blanton, 1959	(Ti, PE, Oz, CE, SD, Ti2)	[49, 54, 56]
	***C.* sp1**	(Ti)	This study
Leoni group	***C. benarrochi*** Ortiz & Mirsa, 1952	(Ti)	This study
	***C. gabaldoni*** Ortíz, 1954		[49, 56]
	***C. glabellus*** Wirth & Blanton, 1956	(Ti, PE)	[49, 56]
	***C. leoni*** Barbosa, 1952		[17, 49, 56]
Limai group	***C. carvalhoi*** Wirth & Blanton, 1973	(Ti)	This study
	***C. limai*** Barretto, 1944	(Ti, PE)	[17, 49, 52, 54, 56]
	***C. lopesi*** Barretto, 1944	(Ti)	This study
	***C. vernoni*** Wirth & Blanton, 1973	(Ti)	This study
	***C.* sp2**	(Ti)	This study
Monticola group	***C. monticola*** Wirth & Lee, 1967 (*pichindensis* Browne, 1980)		[49, 56]
Pachymerus group	***C. pachymerus*** Lutz, 1914	(Ti)	[17]
Reticulatus group	***C. lyrinotatus*** Wirth & Blanton, 1955	(Ti)	This study
	***C. paucienfuscatus*** Barbosa, 1947	(Ti, Ti2)	[56]
	***C. pifanoi*** Ortíz, 1951 *(tricoloratus* Wirth & Blanton, 1953)	(Ti, Oz, Ti2)	[54]
	***C. profundus*** Santarém, Felippe-Bauer & Trindade, 2014	(Ti)	This study
	***C. pseudoreticulatus*** Santarém, Felippe-Bauer & Castellón, 2014	(Ti, Ti2)	This study
Stigmalis group	***C. alvarezi*** Ortíz, 1957		[49, 56]
	***C. fluviatilis*** (Lutz), 1914 (*scorzai* Ortíz, 1956)		[49, 54, 56]

*Andean highlands*: (*Ur*: Urkisiki); *Northern Andean foothills*: (*Pa*: Pacto), (*PE*: Paraiso Escondido), (*SD*: Santo Domingo de los Colorados); *Southern Andean foothills*: (*CE*: Caluma-Echandia); *Amazon Basin*: (*CD*: Cofán-Dureno), (*Ti, Ti2*: Tiputini); *Coast*: (*Oz, Oz2*: Santo Domingo de Onzole) and (Ju: Junin).

¥ Specimens from Ecuador previously considered to be *C. verecundus* were restored from synonymy by Felippe-Bauer *et al.* [15] and are now known *as C. contubernalis* Ortíz & Léon.

The number of *Culicoides* species reported in South America varies by country: 44 species have been reported in Argentina [2], 18 in Bolivia [25], 151 in Brazil [45], 114 in Colombia [56], 31 in Peru [32], and 81 in Venezuela [8].

In Ecuador, biting midges are not monitored as part of a national vector surveillance programme in contrast to European countries that have implemented extensive control strategies in response to BTV emergence (Regulation Commission (EC) No. 1266/2007). Nevertheless, in the last decade, a few local academic studies have contributed to better knowledge of biting midges (particularly in taxonomy and distribution) [19, 30, 31]. We aimed to revise the current inventory of *Culicoides* species present in Ecuador in the light of the recent detection of BTV and OROV. The purpose of this study was to produce a comprehensive list of *Culicoides* species in Ecuador, to provide information that may contribute to implement adequate surveillance and vector control strategies in the future.

## Materials and methods

**Figure 1:**
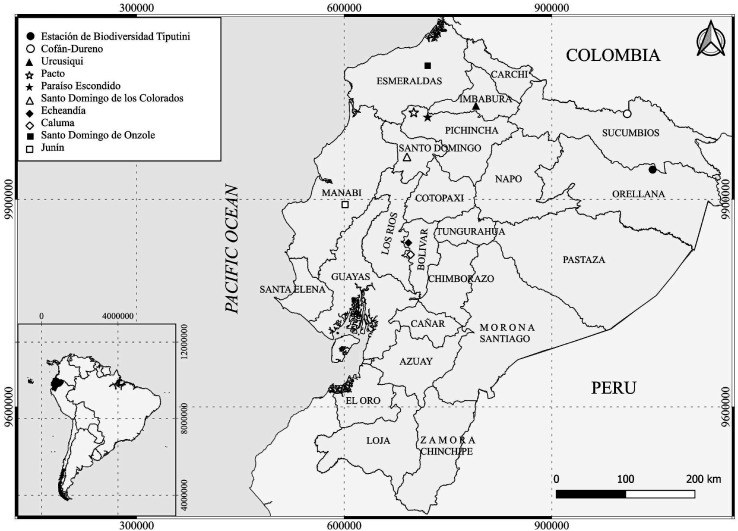
Sites of entomological collections.

Biting midges were captured using CDC light traps between July 2010 and October 2013 and in May 2019 in nine localities from three regions (Fig. 1): The Amazon basin, from Yasuní-Tiputini region (Orellana Province, July 2010 and October 2013) and Cofán-Dureno (Sucumbíos Province, July 2011); the Andean highlands of Urkusiki region (Imbabura Province, September 2012); the northern Andean foothills of Pacto region (Pichincha Province, March 2011), Paraiso Escondido (Pichincha Province, November 2012), Santo Domingo de los Colorados (Santo Domingo de los Tsachilas Province, August 2013), the southern Andean foothills of Caluma and Echeandia (Bolivar Province, March 2013); and the coastal region of Junin (Manabi Province, March 2013) and Santo Domingo de Onzole (Esmeraldas Province, February 2013 and May 2019). This last collection of 2019 was carried out due to the recent report of Oropouche cases in this coastal province. In Orellana Province, research was conducted at Tiputini Biodiversity Station (TBS). TBS is located on the north bank of the Tiputini River, bordering Yasuní National Park and within Yasuní Biosphere Reserve, one of the most diverse regions of the world [4]. Most of the collections were carried out during the dry season (from July to February) since these sites are more accessible.

The collection sites were selected based on the known ubiquitous capture sites of *Culicoides* spp., like animal shelters, forests, and mud rich sites with organic matter [6, 52]. Seven-night catches per site were performed monthly. Traps were set approximately 1 h before sunset until 1 h after sunrise under favorable climatic conditions (absence of heavy rain and/or wind) [1]. All these specimens were stored in 70–100% ethanol. In the laboratory, *Culicoides* specimens were separated from other arthropods using a stereo microscope (Olympus SZ51), placed in 1.5 mL Eppendorf^®^ tubes containing 70% ethanol and stored at −20 °C. Specimens were identified using different morphological keys [13, 15–17, 44, 47, 52, 54, 56, 57]. The *Culicoides* species were classified into subgenera, species groups according to Borkent and Dominiak [7].

Specimen identification was performed, using a microscope, after mounting the head, wings, genitalia and spermathecae on microscope slides with Gum Chloral [1] or Euparal medium [20]. Digital images of the wings were obtained using an Olympus (BX41 or BX53) microscope equipped with an Olympus SC100 camera and software (cellSens or stream). All voucher specimens were deposited in the entomological collections of San Francisco de Quito University, Ecuador.

## Results

**Figure 2:**
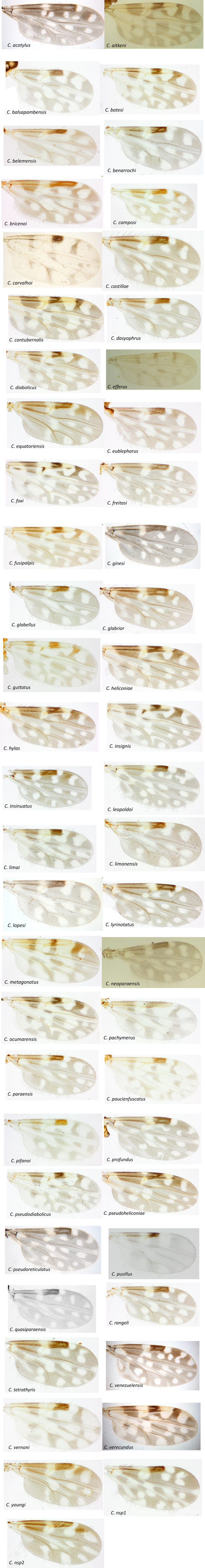
Wing photographs of *Culicoides* species in Ecuador. The new *Culicoides* records are: *C. acotylus*, *C. aitkeni*, *C. benarrochi*, *C. carvalhoi*, *C. freitasi*, *C. ginesi*, *C. lopesi*, *C. lyrinotatus*, *C. profundus*, *C. pseudoreticulatus*, *C. quasiparaensis*, *C. vernoni*, *C. youngi* and two new species (*Culicoides* n. sp. 1 and *Culicoides* n. sp. 2).

Fifty-three species of the genus *Culicoides* were identified morphologically from a total of 12,073 captured *Culicoides* specimens. The locality with the highest abundance was Yasuni-Tiputini in Orellana Province in both collection periods, with 7,691 specimens collected. We also found 73 specimens in Cofan-Dureno, 323 in Pacto, 243 in Paraiso Escondido, 194 in Santo Domingo de los Colorados, 19 in Urkusiki, 1117 in Caluma-Echeandia, 1533 in Santo Domingo de Onzole, and 880 in Junin. Females represent 71.52% of the collected biting midges, while 28.48% were males. We identified 29 species, belonging to seven different subgenera (*Anilomyia* Vargas*, Avaritia* Fox*, Diphaomyia* Vargas, *Haematomyidium* Goeldi*, Hoffmania* Fox*, Mataemyia* Vargas*, Psychophaena* Philippi) (Table 1, Fig. 2). Moreover, we caught 24 species belonging to nine species groups (acotylus, carpenteri, dasyophrus, eublepharus, fluvialis, leoni, limai, pachymerus and reticulatus) (Table 1, Fig. 2). Nine species were found in three Ecuadorian regions (*C. batesi* Wirth & Blanton, *C. castillae* Fox, *C. diabolicus* Hoffman, *C. guttatus* (Coquillet), *C. heliconiae* Fox & Hoffman, *C. hylas* Macfie, *C. insignis* Lutz, *C. pseudoheliconiae* Felippe-Bauer*, C. tetrathyris* Wirth & Blanton). We reported two new species collected in TBS (Table 1). *Culicoides pseudodiabolicus* Fox was only found in the coastal region of Santo Domingo de Onzole (Esmeraldas) in both collection periods. Forty-eight species were caught in the Amazon basin, and 45 of them were collected in TBS. Two species (*C. youngi* Wirth & Barreto, *C. verecundus* Macfie) were found only in the Andean foothills. *Culicoides venezuelensis* Ortíz & Mirsa was the only species found in the Andean highlands. For the distribution of other species, see Table 1.

## Discussion

Fifteen species are herein newly recorded for Ecuador (Table 1 and Fig. 2): *C. aitkeni* Wirth & Blanton, *C. acotylus* Lutz*, C. benarrochi* Ortiz & Mirsa, *C. carvalhoi* Wirth & Blanton*, C. freitasi* Wirth & Blanton, *C. ginesi* Ortíz, *C. lopesi* Barretto*, C. lyrinotatus* Wirth & Blanton, *C. profundus,* Santarém, Felippe-Bauer & Trindade, *C. pseudoreticulatus* Santarém, Felippe-Bauer & Castellón*, C. quasiparaensis* Clastrier, *C. vernoni* Wirth & Blanton, *C. youngi* Wirth & Barreto and two new species; one belongs to the fluvialis group according to DNA barcoding [1] and the other belongs to the limai group according to morphological characters.

Additionally, we detected 13 species that have been reported previously only once in Ecuador (Table 1): *C. efferus* Fox*, C. belemensis* Wirth & Blanton*, C. foxi* Ortiz, *C. guttatus* (Coquillett), *C. hylas* Macfie, *C. insignis*, *C. leopoldoi* Ortiz*, C. neoparaensis* Tavares & Souza, *C. pachymerus* Lutz, *C. paucienfuscatus* Barbosa*, C. pifanoi* Ortíz, *C. pseudoheliconiae* Felippe­Bauer and *C. venezuelensis* Ortiz & Mirsa. *Culicoides venezuelensis* was found for the first time in the Ecuadorian Amazon basin (Sucumbios province) and Andean highlands (Imbabura province). *Culicoides batesi*, *C. castillae*, *C. diabolicus*, *C. guttatus*, *C. heliconae*, *C. hylas*, *C. insignis*, *C. pseudoheliconiae* and *C. tetrathyris* were found in three regions (Coast, Andean, and Amazon basin). The highest number of species was found in the Amazon basin, in the Tiputini region. This finding corroborates other studies that suggest that Tiputini harbors immense biodiversity and probably the highest diversity of insects in the world (100,000 species/ha) [4, 59]. Our results increase the number of Ecuadorian *Culicoides* species to 70 (Table 1).

*Culicoides insignis, C. pachymerus*, and *C. paraensis* have medical and/or veterinary importance because they have been reported as possible causative agents of dermatozoonosis [42, 46]. Intriguingly, three species identified in this study (*C. foxi*, *C. insignis,* and *C. filarifer*) have been reported to carry DNA from *Leishmania brasiliensis* and *Le. Amazonensis* [39]. Additionally, *C. foxi* is a causative agent of allergic dermatitis and *C. pifanoi is* known as a causative agent of filariasis [8]. Moreover, *C. acotylus*, *C. fluvialis*, and *C. leopoldoi* are suspected to cause allergic dermatitis in humans [8]. Finally, *C. guttatus* may play a role as a vector of mansonellosis, a neglected tropical disease [38] and of BTV [8]. Further investigations are needed to determine the role of these species in dermatological disorders.

According to [6, 10, 45], the reported distribution of the species newly cited herein for Ecuador are: i) *C. acotylus* in Brazil (Amazonas, Pará, Rondônia, Roraima, and Mato Grosso), Honduras, Mexico, Panama, Suriname, Trinidad and Tobago, and Venezuela; ii) *C. aitkeni* in Brazil (Amazonas, Pará); Trinidad and Tobago; iii) *C. benarrochi* in Brazil (Amazonas, Roraima, and Rio de Janeiro), Trinidad and Tobago and Venezuela; iv) *C. carvalhoi* in Brazil (Pará); v) *C. freitasi* in Brazil (Amazonas and Pará); vi) *C. ginesi* from El Salvador to Panama*,* northeastern Argentina, Brazil (Pará, Rondônia), Colombia, Trinidad and Tobago and Venezuela; vii) *C. lopesi* in Brazil (Acre, Amazonas, Rio de Janeiro, and São Paulo), Panama and Suriname; viii) *C. lyrinotatus* in Brazil (Pará), Nicaragua and Panama; ix) *C. profundus* in Brazil (Amazonas, Pará and Rondônia); x) *C. pseudoreticulatus* in Brazil (Amazonas, Rondônia and Roraima); xi) *C. quasiparaensis* in Brazil (Acre, Amazonas, Pará, Rondônia, Roraima, and Maranhão), Colombia, El Salvador, French Guiana, Honduras and Peru; xii) *Culicoides vernoni* in Brazil (Amazonas, Rondônia and Pará), Bolivia, Colombia and Costa Rica; and xiii) *C. youngi* in Brazil (Amazonas) and Colombia.

*Culicoides paraensis* is distributed from the USA to Argentina [49, 56], and it is abundant in the humid tropics in Panama [52] and in urban areas with banana crops in the Amazon region and in the southern states of Brazil [14]. It was collected in South Carolina zoos [33]. In Peru, populations of *C. paraensis* seemed to be highest from October to December, with numbers fluctuating depending on the year and collection site [29]. Interestingly, the presence of *C. paraensis*, one of the known vectors of OROV [40], was also confirmed herein in Ecuador. An estimated half-million people have been affected by OROV since it was first isolated in Brazil [9]. In Ecuador, OROV was detected in the Esmeraldas Province (Coast) [58] and there is serological evidence in febrile patients from the Amazon basin (Pastaza Province) [26]. In our study, *C. paraensis* was found in Orellana province, located north of Pastaza province, suggesting that this vector may be transmitting OROV in Pastaza province where serologic evidence was detected [26]. In contrast, *C. paraensis* was absent in the Coastal region, (Esmeraldas and Manabi Provinces). Future investigations will address the distribution of this species particularly in forested and urban areas in Ecuador. In this study, *C. neoparaensis*, *C. paraensis*, and *C. quasiparaensis* were collected in the same area (Yasuní-Tiputini – Amazon basin). These three species belong to the paraensis group that includes seven species (*C. diversus* Felippe-Bauer, *C. filiductus* Wirth, *C. peruvianus* Felippe-Bauer, and *C. austroparaensis* Spinelli) [13, 48]. It is worth remarking that our identifications were based only on morphological characters; thus, further molecular analysis would be needed to explore the boundaries of these closely related/similar species.

Finally, *C. insignis* is considered the main candidate vector species of BTV in South America [5], an arbovirus that infects livestock worldwide and is responsible for global losses of agriculture of up to US$3 billion [41]. Bluetongue virus has been detected in blood samples from Ecuadorian cattle [11, 50]; however, its vector species in Ecuador have not been confirmed yet. *Culicoides insignis* is distributed in the USA (Alabama, Georgia, and Florida states), Mexico (Chiapas, Yucatán), Central America and Caribbean to central Argentina [6, 49, 56]. It is abundant in the arid tropics and absent in humid tropics in Panama [52]. Populations of *C. insignis* were highest from July to December at most sites in the Caribbean, and from June to October in Central America [18]. Therefore, in this region, nulliparous and parous females of this species were captured throughout the year [18]. In this study, *C. insignis* was collected in the Amazon basin and in the foothills (from north to south) of the Andean region until 2013, but we also found specimens in 2019 in the coastal region of Esmeraldas province; thus, it is possible that this species has favorable adaptation capacities due to its broad distribution throughout South America [3, 12].

In conclusion, our examination of fresh material revealed 15 new species records for the country, and confirmed 13 species previously reported in Ecuador. Herein, we present an updated list of the Ecuadorian *Culicoides* that comprises 70 species, including possible vectors of human and veterinary interest. This species inventory is a prerequisite for the future development of a barcode library and the construction of an image database of *Culicoides* wings. Both may be useful for further ecological studies and to establish risk maps for *Culicoides*-borne diseases in Ecuador.
